# Prospects for fertility preservation: the ovarian organ function reconstruction techniques for oogenesis, growth and maturation *in vitro*

**DOI:** 10.3389/fphys.2023.1177443

**Published:** 2023-05-12

**Authors:** Bai Hu, Renjie Wang, Di Wu, Rui Long, Jinghan Ruan, Lei Jin, Ding Ma, Chaoyang Sun, Shujie Liao

**Affiliations:** ^1^ Department of Gynecological Oncology, Tongji Hospital, Tongji Medical College, Huazhong University of Science and Technology, Wuhan, China; ^2^ National Clinical Research Center for Obstetrics and Gynecology, Cancer Biology Research Center (Key Laboratory of the Ministry of Education), Tongji Hospital, Tongji Medical College, Huazhong University of Science and Technology, Wuhan, China

**Keywords:** ovarian organ function reconstruction techniques, fertility preservation, tissue engineering, *in vitro* culture of oocytes, artificial ovary, organoids, artificial oocytes, reproduction

## Abstract

Today, fertility preservation is receiving more attention than ever. Cryopreservation, which preserves ovarian tissue to preserve fertility in young women and reduce the risk of infertility, is currently the most widely practiced. Transplantation, however, is less feasible for women with blood-borne leukemia or cancers with a high risk of ovarian metastasis because of the risk of cancer recurrence. In addition to cryopreservation and re-implantation of embryos, *in vitro* ovarian organ reconstruction techniques have been considered as an alternative strategy for fertility preservation. *In vitro* culture of oocytes in vitro Culture, female germ cells induction from pluripotent stem cells (PSC) *in vitro*, artificial ovary construction, and ovaria-related organoids construction have provided new solutions for fertility preservation, which will therefore maximize the potential for all patients undergoing fertility preservation. In this review, we discussed and thought about the latest ovarian organ function reconstruction techniques *in vitro* to provide new ideas for future ovarian disease research and fertility preservation of patients with cancer and premature ovarian failure.

## 1 Introduction

Nowadays, with the development of malignant tumors at a younger age (Gershenson, 2019), chemotherapy and radiotherapy have immediate and long-term side effects on ovarian function. The first effect of chemotherapy on the ovarian is immediate. It is cytotoxicity to dividing cells, which may directly kill growing follicles and induce premature ovarian failure (POF). Chemotherapy may also induce inflammation and destruction of vascular and stroma, which is harmful to the growth of the follicle. Moreover, the acute decrease in growing follicles, which leads to the reduction of sex steroid hormones and inhibin, may activate primordial follicles, enhance the rate of recruitment, accelerate the depletion of the reserve, and finally lead to POF (Sciorio and Anderson, 2020). The frequency of POF after radiotherapy is related to the used dose of radiation. Whole irradiation doses at 3–5 Gy, 60% of the follicles are destroyed; with irradiation at doses of 5 Gy, 100% of the follicles are destroyed (Dinas, 2020). To meet reproductive and endocrine needs, cryopreservation of ovarian tissue for transplantation before starting anti-cancer treatment is the main means of fertility preservation. However, this method has the risk of reintroducing malignant tumor cells. For patients with ovarian diseases such as POF, the search for safe and efficient fertility preservation methods is also of great importance. In order to solve this problem, the ovarian organ function reconstruction techniques for oogenesis, growth and maturation *in vitro* are gradually becoming the core of research.

**FIGURE 1:**
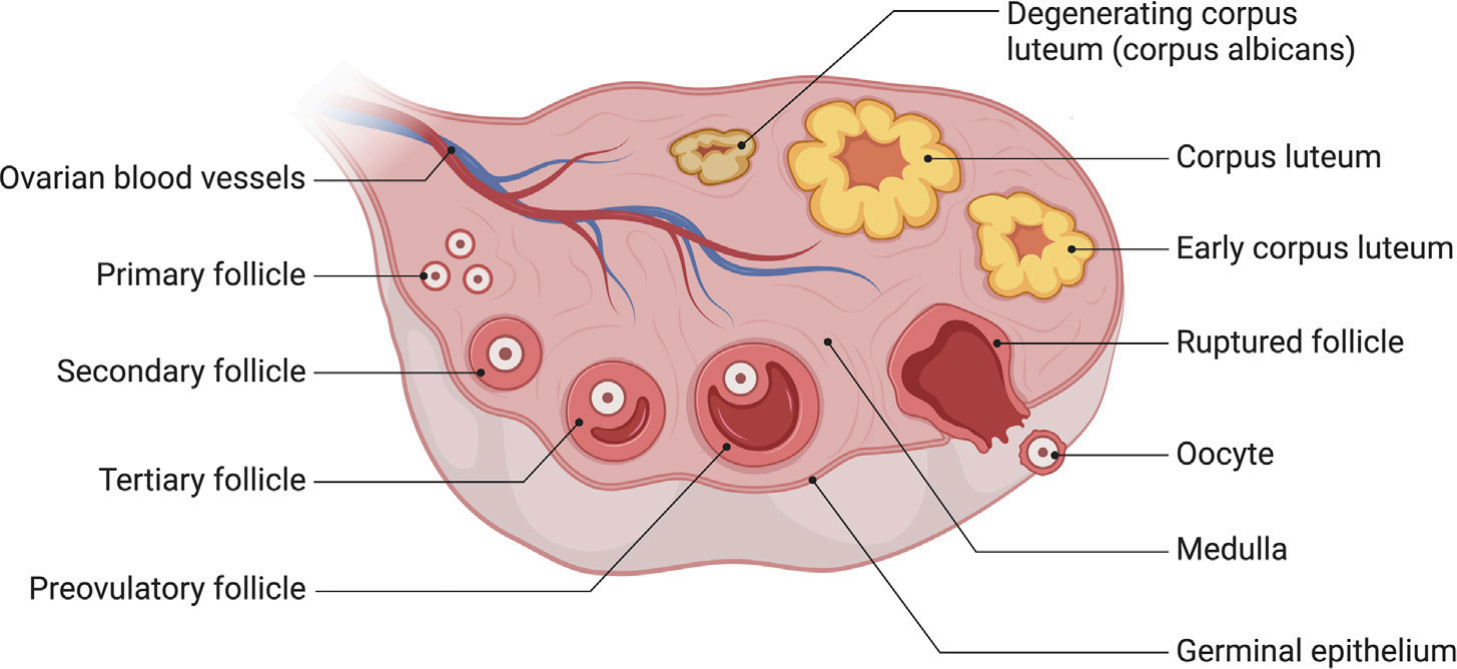
The anatomy of human ovary. The cortex is the principal part of the ovary, containing various levels of developing follicles, corpus luteum, interstitial tissue, *etc.*, whereas the medulla is mainly composed of blood vessels, nerves, lymphatics, and loose connective tissue. Created with BioRender.com.

Histologically, human ovarian germinal epithelium (OSE) is divided into the outer cortex and the inner medulla. The cortex is the principal part of the ovary, containing various levels of developing follicles, corpus luteum, interstitial tissue, *etc.*, whereas the medulla is mainly composed of blood vessels, nerves, lymphatics, and loose connective tissue (Frances-Herrero et al., 2022) (Figure 1).

Follicles are the primary functional units of mammalian ovaries. In most mammals, follicle development starts before birth and continues throughout the reproductive years. Generally, the development of the follicle includes three processes:1) primordial follicle is activated and grows to the preantral follicle stage; 2) preantral follicle goes through antral follicular stage to preovulatory follicle as follicular fluid increases to form a follicular cavity; 3) due to the effect of luteinizing hormone/follicle-stimulating hormone releasing hormone (LH/FSH) peak, preovulatory follicle releases oocyte corona cumulus complex (OCCC) and oocyte (Telfer and Zelinski, 2013).

**FIGURE 2:**
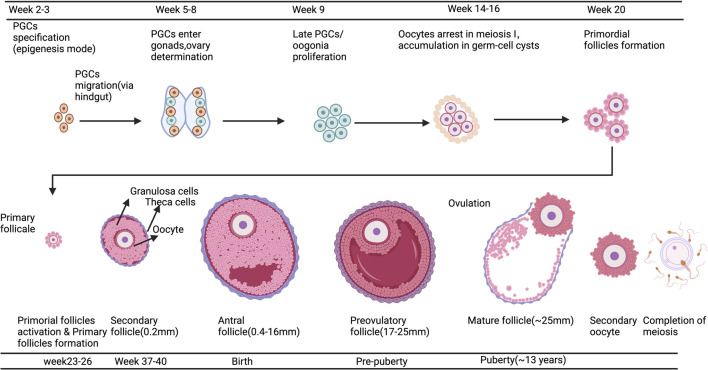
A Schematic comparison of developing germ cells, oocytes, and ovarian follicles in humans. The primordial germ cells (PGCs) are specified by the epigenesis mode in humans. After specification, PGCs migrate to the gonads; this occurs via the hindgut in humans. Further physiological events in the differentiated ovary, including oogonia proliferation and entry into meiosis, oocyte arrest in meiotic prophase I, the accumulation of oocytes in germ-cell cysts, and the formation of early follicles, are all essentially the same in humans. Created with BioRender.com.

The process of human oocyte growth and development is continuous and complex: during early embryonic development, primordial germ cells (PGCS) actively migrate to the gonads, undergo pluripotency recapture, epigenetic reprogramming (including histone modifications, X-stained weight activation, genome-wide DNA demethylation, and blot erases) and sexual differentiation to form oogonia. Finally, Meiosis II(MII) oocytes are generated through Meiosis, and the second Meiosis is completed when spermatozoa meet (Garcia-Alonso et al., 2022) (Figure 2). Oocyte development and growth depend on the follicular environment; as a result, the ovarian organ function reconstruction system should promote follicle activation, growth, and maturity or provide a follicle-like microenvironment for oocyte development. It will allow complex dynamic bidirectional communication between the oocyte and surrounding granulosa cells and follicle theca cells, completing the critical process of oocyte meiosis. By establishing a safe and effective ovarian organ rebuilding system, it will be feasible for oogenesis, growth and maturation *in vitro* to make the most of the ovarian follicular reserve and even restore fertility in the absence of ovarian follicle reserves.

Recently, the ovarian organ function reconstruction techniques for oogenesis, growth and maturation *in vitro* mainly include *in vitro* follicle culture (IVC), induction using pluripotent stem cells (PSCs) *in vitro*, artificial ovary construction technology and ovarian organoids. In addition, tissue engineering techniques, which combine biology, medicine, and materials engineering (Langer and Vacanti, 2016), are also being extensively studied to reconstruct or repair ovarian structures. In order to provide fresh perspectives on the preservation of patients’ fertility who have cancer, POF, *etc.*, as well as on future research on ovarian diseases, this article reviews the most recent techniques for ovarian organ function reconstruction techniques for oogenesis, growth and maturation *in vitro*.

## 2 Follicle *in vitro* culture (IVC)

**FIGURE 3:**
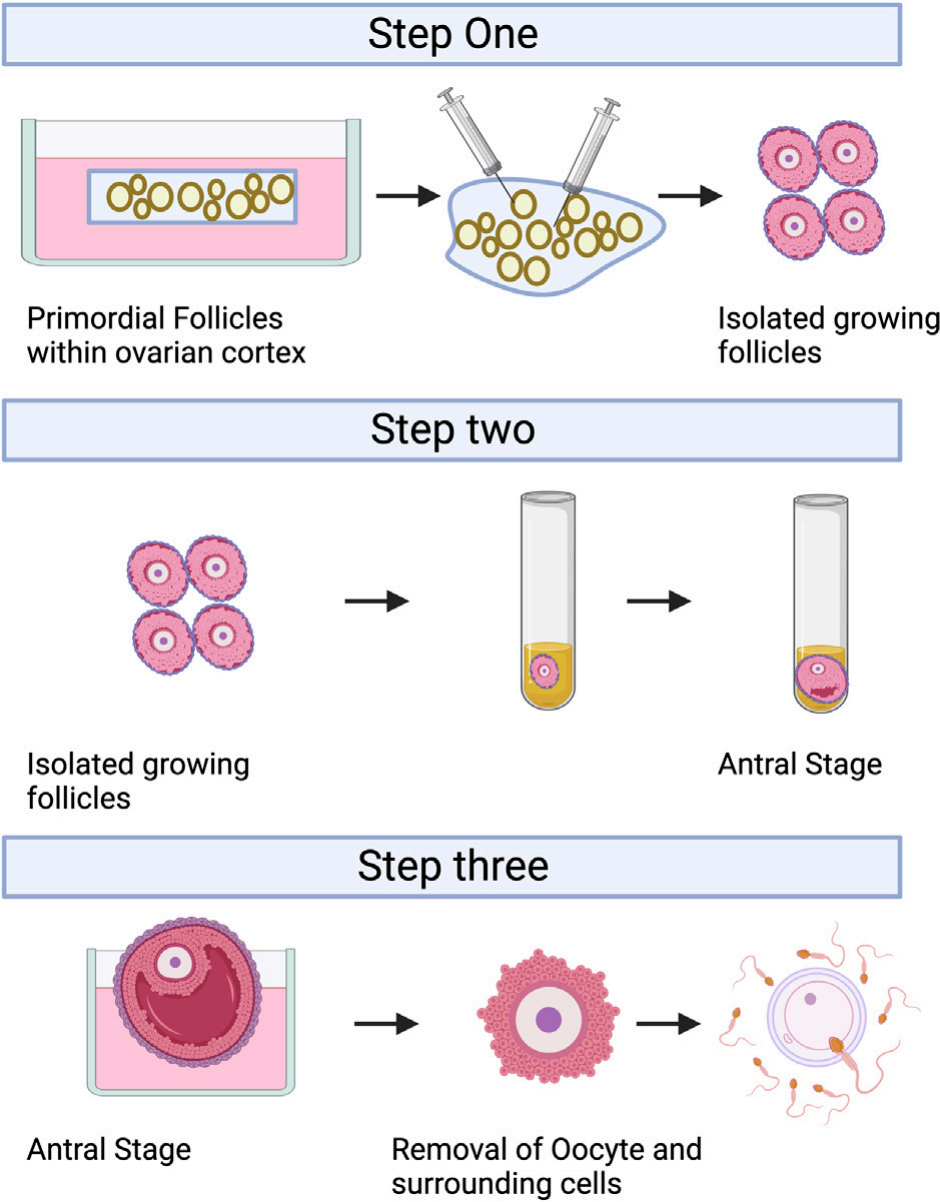
A multistep culture system to support *in vitro* growth (IVG) of oocytes from human primordial follicles through to maturation. Step one: Flattened strips of ovarian tissue are cultured free-floating in medium containing human serum albumin, ascorbic acid, and basal levels of follicle-stimulating hormone. Once follicles have reached multilaminar stages, they can be isolated mechanically using needles. Step two: Isolated follicles are cultured individually from the preantral to the antral stages. Step three: The final stages of oocyte growth and development are achieved by removing the oocyte-cumulus complex from the antral follicle and culturing the oocyte and its surrounding somatic cells. Oocyte-cumulus complexes are placed within medium for *in vitro* maturation (IVM). Oocytes are then analyzed for the presence of a metaphase II spindle and a polar body. Created with BioRender.com.

IVC refers to the isolation of immature follicles from ovarian tissue, *in vitro* culture to maturity, and subsequent *in vitro* fertilization (IVF), which can avoid the risk of tumor reimplantation and utilize a large number of immature follicles in the ovary. The follicular multistage IVC system has been applied into practice in many species: in mice, it can be cultured from primordial follicles to live birth (Mochida et al., 2013); In primates, it can be cultured from secondary follicles to morula stage (Xu et al., 2013); and in humans, there have been reports of MII stage oocytes cultured from primary or secondary follicles (Xu et al., 2021). However, compared with humans, the prolonged growth period of complete follicular formation in mice, larger follicles in humans, and different effects of growth factors and hormones between species are factors that hinder the cross-species translation of these technologies (Herta et al., 2018; Telfer, 2019a; Telfer, 2019b; Telfer et al., 2019). The follicular multistage IVC system is mainly carried out in three steps: 1) Culturing the small ovarian cortex to support primordial follicle activation (*in vitro* activation, IVA) and early growth and supporting its early growth; 2) Isolating and culturing growing presinus follicles to achieve oocyte growth and development to the sinus stage (*In vitro* growth, IVG); 3) *In vitro* maturation (IVM) of oocytes (Yang et al., 2020a). Figure 3 shows the whole multistep culture system to support *in vitro* growth of oocytes from human primordial follicles through to maturation. The ovarian environment is dynamic, controlled by cyclical changes in the receptor cycle of hormones and local signals from the ovary itself. The significant principle of using tissue engineering to construct IVC system is to accurately present the changes of various signals (including growth factors, hormones, extracellular matrix (ECM), mechanics, *etc.*), so as to allow the coordinated growth of multiple cell compartments (oocytes and surrounding granulosa cells, follicular theca cells) in the follicle. When simulating the ovarian environment *in vitro,* the follicles must be provided with signals such as growth factors and hormones at appropriate times and concentrations. Therefore, it can promote cell growth and development and promote cell-cell communication, maintain the complex interaction between oocyte and ovarian somatic cells (granulosa cells and follicular theca cells), and meet the changing needs of oocyte and ovarian somatic cells.

### 2.1 Follicle *in vitro* activation (IVA)

Activation of primordial follicles is a key initial step in oocyte development. In females, dormant primordial follicles consist of a single layer of primordial follicle granulosa cells (pfGCs) wrapped around primary oocytes, and the dormant state is influenced by forkheadbox O3 (FOXO3), the phosphatase and tensin homolog deleted on chromosome 10(PTEN), TSC complex subunit 1 (TSC1), TSC complex subunit 2 (TSC2), forkheadbox L2(FOXL2), p27KIP1 gene (p27), ribosomal protein S6 (rpS6), 3-phosphatidylinositol-dependent protein kinase 1 (PDK1), and anti-mullerian hormone (AMH) (Reddy et al., 2010; Chen et al., 2020), *etc.* When the regulation of certain factors is altered, the dormant state of the primordial follicle is broken and thus activated to continue development, or degeneration or atresia occurs. IVA can be used to activate growth before transplantation to cause a burst of sinus follicle development 4–6 months later, from which oocytes can be harvested, which would benefit women with no ovarian reserve or who produce only a small number of follicles even after intense ovarian stimulation (Kawamura et al., 2013; Suzuki et al., 2015; Zhai et al., 2016). Notably, when human primordial follicles are isolated from human ovarian cortical tissue, the survival rate of culturing isolated primordial follicles is extremely low (Hovatta et al., 1997; Oktay et al., 1997); therefore, primordial follicle-based cultures need to incorporate both human ovarian cortex and not isolated primordial follicles. Because mechanical signals modulate the activation pathway, ovarian cortical tissue containing primordial follicles was activated more quickly in flat “sheets” culture than in cube culture (Yding Andersen et al., 2019).

Currently, the commonly used IVA basal media mainly include α-MEM, D-MEM, Waymouth and McCoys 5a (Yang et al., 2020a), and researchers alter culture environment by modulating key pathways and adding small molecule compounds to improve IVC follicle number and quality.

**FIGURE 4:**
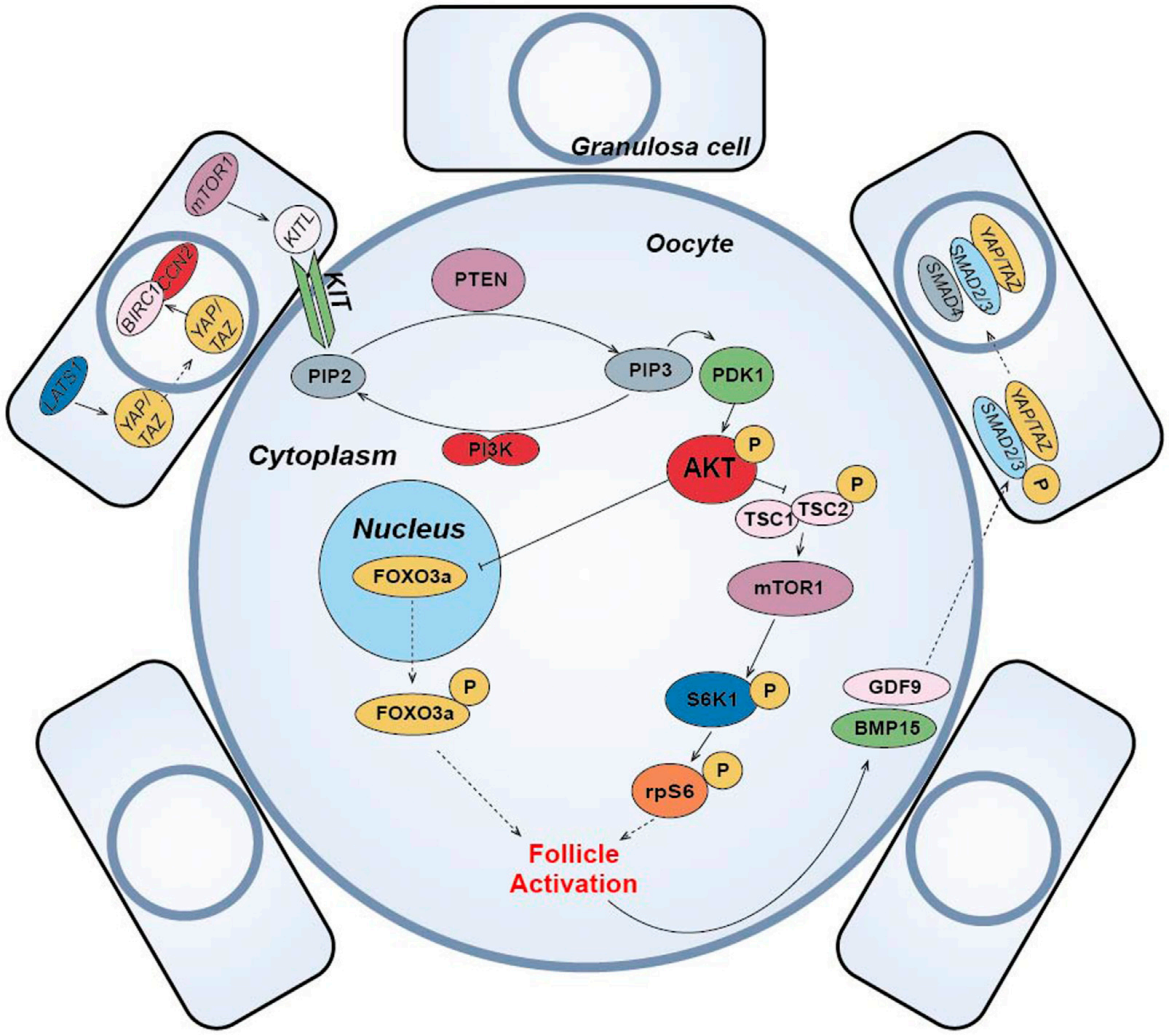
Schematic diagram of primordial follicle activation signaling pathway. The “p” represents phosphorylation, the solid arrow represents activation, the solid T-shaped arrow represents inhibition, and the dotted arrow represents entering or exiting the nucleus or cell membrane.

(1) The PI3K-PTEN-AKT-FOXO3 signaling pathway is the main non-gonadotropin growth factor signaling pathway that has been agreed upon to regulate ovarian follicle growth and differentiation (Reddy et al., 2008; Li et al., 2010; Grosbois and Demeestere, 2018; Maidarti et al., 2019) (Figure 4). PI3K, consisting of a regulatory subunit (p85) and a catalytic subunit (p110), is a key factor in the reception of granulosa cell signaling in the primary oocyte membrane (Hsueh et al., 2015). PI3K-mediated signaling converges at PDK1 (Telfer and Zelinski, 2013), where PI3K in the primary oocyte membrane is stimulated by the upstream receptor complex kinase to phosphorylate phosphatidylinositol 4,5-bisphosphate (PIP2) to phosphatidylinositol 3,4,5-trisphosphate (PIP3), while PDK1 transduces the signal to free intracellular Akt, which is stimulated to phosphorylate and transmit the signal from the cell membrane to the nucleus. The follicular dormancy factor Foxo3a in the nucleus is inhibited by receiving signals from Akt and moves out of the nucleus (Hsueh et al., 2015). Upon release from the dormant inhibition of Foxo3a, primary oocytes are activated and continue to develop, thus inducing the activation of the initiating follicle development. Among them, PTEN plays a negative regulatory role in this signaling pathway and is involved in the activation and dormancy regulation of the initiating follicle by dephosphorylating PIP3 to PIP2, and inhibition of PTEN expression has a facilitative effect on the activation of the initiating follicle (Hsueh et al., 2015; Novella-Maestre et al., 2015). In 2013, scientist Kazuhiro Kawamura reported for the first time that *in vitro* addition of PI3K activator 740Y-P and PTEN inhibitor bpV (HOpic) to human ovarian tissue successfully activated the follicles of POI patients to achieve live births, opening a new paradigm of IVA for infertility treatment (Kawamura et al., 2013). Notably, more than 50% of the POI patients in this study did not contain residual follicles and did not respond to IVA drugs, limiting the use of IVA procedures in infertility treatment. In addition, PTEN inhibition affects ovarian DNA repair (Martin et al., 2019), and a process to study IVC in bovine ovarian cortex showed that PTEN inhibition activated non-growing follicles in cattle, but simultaneously increased follicular DNA damage and decreased DNA repair responses (Maidarti et al., 2019). Recently, a drug-free IVA approach has led to successful pregnancies in POI patients (Ferreri et al., 2020), which eliminates the negative effects of activators on follicles, but may also reduce follicle production sufficiently for subsequent culture.
(2) In pfGCs, mTORC1 is a key factor in the reception of follicle activation signals (Zhang et al., 2014) (Figure 4). PfGCs induce primary oocyte activation through signaling via the mTORC1-KIT ligand (KITL) signaling pathway to dormant primary oocytes (Zhang and Liu, 2015). When pfGCs are affected by changes in nutrition, oxygen concentration and growth factors, mTORC1 activity on pfGCs is enhanced and further stimulates KITL, resulting in upregulation of KITL secretion. KIT receptors on primary oocytes bind to secreted KITL and continue to stimulate PI3K in primary oocytes (Zhang and Liu, 2015), thereby activating the PI3K/PTEN/Akt signaling pathway. In primary oocytes, mTORC1 is also a regulator of dormancy and activation and is negatively regulated by a heterodimeric complex composed of the tumor suppressors Tsc1 and Tsc2 (Reddy et al., 2010). When Akt inhibits the TSC1/2 complex and subsequently inhibits downstream mTORC1 expression, resulting in reduced mTORC1 activity, the inhibited mTORC1 promotes phosphorylation of the downstream ribosomal protein S6 Kinase 1 (S6K1), which continues to stimulate downstream rpS6 and subsequently promotes primary oocyte protein translation and ribosome genesis (Adhikari et al., 2010), initiating primary oocyte development. Moreover, it has been suggested that mTORC2 is also involved in regulating Akt and thus mediating the downstream activation mechanism (Sarbassov et al., 2005).
(3) Hippo signaling pathway can be activated by cutting ovarian tissue under *in vitro* culture conditions to induce follicle activation and early development (Figure 4). In 2018, the results of J. Grosbois et al. confirmed that in chopped cultured ovarian tissue, more follicles were activated at the margins of the tissue than at the middle of the tissue and that the activation mechanism was due to the disruption of the Hippo signaling pathway by inhibition (Grosbois and Demeestere, 2018). In dormant primordial follicles, Hippo signaling pathway-mediated activation mechanisms take place mainly in pfGCs. When ovarian tissue is fragmented, contact inhibition between pfGCs at the tissue margin is released and expression of large tumor suppressor gene 1 (LATS1) in the Hippo signaling pathway increases. Highly expressed LATS1 inhibits phosphorylated YES-associated protein (YAP)/PDZ-binding domain transcriptional co-activator (TAZ) specifically expressed by pfGCs, allowing its translocation into the nucleus. Accumulation of YAP/TAZ in the nucleus enhances transcription of connective tissue growth factor (CTGF/CCN2) and baculovirus repetition 1 protein (BIRC1), both of which promote the development of pfGCs (Pan, 2007; Herta et al., 2018). The developed pfGCs then transmit signals to primary oocytes through the mTORC1-KITL signaling pathway and induce activation. After activation, GDF9 and BMP15 secreted by primary oocytes can coordinate with YAP in pfGCs to promote the phosphorylation of receptor-regulated SMAD2/3 protein. Then, it binds with co-modulating SMAD4 protein to form AP-Smad2/3/4 complex into the nucleus, which can also promote the development of pfGCs (Grosbois and Demeestere, 2018). In conclusion, Hippo signaling can cooperate with PI3K/PTEN/Akt signaling to promote the activation and development of dormant primordial follicles (Grosbois and Demeestere, 2018). Disruption of Hippo signaling pathway-mediated follicle activation has the advantage of using only physical cutting methods without the addition of other chemicals to achieve *in vitro* activation of dormant primordial follicles, effectively reducing the *in vitro* culture time of ovarian tissue and improving the safety of the IVA technique. Usually, the regulation of PI3K/PTEN/Akt signaling pathway is accompanied by the activation of Hippo signaling pathway when implementing IVA technology, which synergistically promotes the effect of IVA.
(4) In addition to the important role of the above pathways in IVA, some studies have found that the Wnt/β-catenin signaling pathway is not only closely related to embryonic development, organogenesis and disease occurrence (Clevers and Nusse, 2012), but also related to the development and regulation of follicular granulosa cells in mammalian ovarian tissue (Li et al., 2014) (Figure 4). Wnt/β-catenin plays a role in the dormancy and activation of mammalian primordial follicles by mediating Foxo3a expression (Li et al., 2014). Like Foxo3a, p27 gene also plays a dormancy inhibitory role in primordial follicle regulation, but p27 gene and Foxo3a are regulated independently. Only when p27 gene is absent, primordial follicle is activated through PI3K/PTEN/Akt/p27 pathway (Zhang and Liu, 2015). Other studies have suggested that glycogen synthetase kinase 3 (GSK-3) also regulates primordial-follicular activation and development through the PI3K/PTEN/Akt/GSK-3 pathway (Liu et al., 2007). By stimulating mitogen activated protein kinase 3/1 (MAPK3/1), the development of primordia follicles was also activated through the mTORC1-KITL pathway (Zhao et al., 2018). E.H. Ernst et al. also analyzed the development process of human primordia follicles to primary follicles by transcriptome, and found that FOXL2 gene and FOG2 gene expression changed significantly. This indicates that FOXL2 and FOG2 genes also play important roles in the regulation of human primordial follicle activation (Ernst et al., 2018) (Figure 4).
(5) Other small molecule compounds similarly act to stimulate primordial follicle development:In α-MEM basal medium, the addition of human albumin and ITS (insulin, transferrin, selenium) is beneficial to the activation and growth of human follicles and leaves fewer atretic follicles. Meanwhile, the addition of FSH can greatly reduce the number of atretic follicles while increasing the diameter of follicles (Wright et al., 1999). Anti-mullerian hormone (AMH) may affect initiation follicle activation and follicle growth in a dose-dependent manner. Studies have found that the addition of 300 ng/mL AMH in the culture medium can promote the recruitment and activation of human follicles (Schmidt et al., 2005), while the addition of 100 ng/mL AMH inhibits follicle activation (Carlsson et al., 2006). Members of the TGF-β superfamily, such as growth differentiation factor-9 (GDF-9) and bone morphogenetic protein-15(BMP-15) (Sanfins et al., 2018) (**Figure 4**) were found to affect the cell communication between oocytes and somatic cells and promote the activation of human primordial follicle (Kedem et al., 2011). In addition, basic fibroblast growth factor (bFGF), keratinocyte growth factor (keratinocyte growth factor), KGF, leukemia inhibitory factor (LIF), stem cell factor (SCF), vascular endothelial growth factor (VEGF), VEGF and cyclic adenosine monophosphate (cAMP) have both been proved that can improve the activation and survival of cultured follicles *in vitro* (Asadi et al., 2017; Bertoldo et al., 2018). Shown in Table 1.

**TABLE 1:** Additives and Operation in human follicle in IVA stage.

Additives/Operation	Dose	Pathway/Mechanism	Effect	Author
740Y-P	150 μg/mL	PI3K/AKT activator	More follicles activated, no survival difference; Higher serum AMH	Grosbois et al. (Grosbois and Demeestere, 2018); Suzuki N et al. (Suzuki et al., 2015)
				Kawamura K (Kawamura et al., 2013) et al
bpV(HOpic)	30 μM	PTEN inhibitor	More follicles activated	Grosbois et al. (Grosbois and Demeestere, 2018); Suzuki N et al. (Suzuki et al., 2015)
				Kawamura K (Kawamura et al., 2013) et al
Cutting ovarian tissue	—	Hippo signaling pathway	Reduce the *in vitro* culture time of ovarian tissue and improve the safety of the IVA technique	Grosbois et al. (Grosbois and Demeestere, 2018)
rhAMH^a^	300 ng/mL	May rescue some follicles from entering atresia	Enhance follicles recruitment, survival and growth	Schmidt KL et al. (Schmidt et al., 2005)
rrAMH^b^	100 ng/mL	Act as a negative paracrine feedback signal	Suppress the initiation of the growth of primordial follicles, without detrimental effect on viability or follicle density	Carlsson IB et al. (Carlsson et al., 2006)
FSH	300mIU/mL	Prevent apoptotic atresia; Mitogenic function	Reduce the proportion of atretic follicles; Increase follicle size and healthy follicles	Wright CS et al. (Wright et al., 1999)
HAS + ITS	2.5% HSA; 1% ITS^c^	Promote cell proliferation; Act as free-radical scavengers	Reduce the proportion of atretic follicles; Increase follicle size and healthy follicles	Wright CS et al. (Wright et al., 1999)
GDF9	10 ng/mL	BMP15 activates the intracellular signal-mediated pathways Smad1, Smad5, and Smad8	More follicles activated; More follicles activated; increase PCNA expression	Kedem A et al. (Kedem et al., 2011)
	100 ng/mL			
BMP15	10 ng/mL	GDF9 activates Smad2 and Smad3	More follicles activated; increase PCNA expression	Kedem A et al. (Kedem et al., 2011)
	100 ng/mL			

^a^ rhAMH:recombinant human anti-Mullerian hormone.

^b^ rrAMH: recombinant rat anti-Mullerian hormone.

^c^ HSA, human serum albumin; 1% ITS:10 mg/mL insulin, 5.5 mg/mL transferrin, and 6.7 ng/mL sodium selenite.

### 2.2 Follicle *in vitro* growth (IVG)

Once the follicle reaches the secondary follicle stage with multiple layers of granulosa cells, it needs to be separated from the cortex, as this would otherwise inhibit its further development (Telfer et al., 2008; Anderson et al., 2014; McLaughlin et al., 2018). Mechanical separation by fine syringe needles is now commonly used (Spears et al., 1994; Cortvrindt et al., 1996; McLaughlin et al., 2018), and this method is commonly used to obtain secondary follicles from mouse ovarian tissue, preserving the complete follicular morphology including an intact theca cell layer and a normal basement membra. However, this method is time-consuming and the production of mature follicles obtained by this method is relatively low. In order to improve the efficiency of follicle separation and for higher density of ovarian tissues such as human and bovine, the efficiency of follicle acquisition can now also be improved by enzymatic methods or a combination of enzymatic and mechanical methods (Telfer et al., 2000). Enzymes such as collagenase and DNase are commonly used to isolate growing follicles from the ovarian cortex, but these enzymes may damage follicular theca cells and may be detrimental to follicle morphology and survival (Telfer et al., 2000; Telfer et al., 2008; Kim et al., 2018; Yang et al., 2020b). However, methods such as improving the purity of enzymes can reduce the damage observed after treatment with collagenase (Dolmans et al., 2006; Rice et al., 2008). A recent study comparing the isolation efficiency of four isolation methods (two mechanical and two enzymatic methods) applied to secondary follicles in mice concluded that the mechanical method had better follicle growth, survival and MII rates, while the method using a cell isolation kit (Sigma Aldrich) was more efficient than traditional mechanical isolation (Kim et al., 2018). In general, each method is unique and requires a carefully designed isolation method based on differences in ovarian tissue of different species.

After isolation of secondary follicles, it is challenging to grow them while maintaining their normal structural state. In the previous multi-step IVC system for human follicles, the isolated secondary follicles were placed directly in 96-well V-bottomed plates without any extracellular matrix or scaffold, and the serum-free medium contained low doses of follicle stimulating hormone (FSH), activin-A, and ascorbic acid (Telfer et al., 2008; Anderson et al., 2014; McLaughlin et al., 2018). 62% of follicles completed the process of growth, differentiation and follicular antrum formation, but only 8% of follicles eventually reached the MII stage (McLaughlin et al., 2018). Growing human antral follicles to preovulatory size *in vivo* is technically challenging, and maintaining the growth of multilayered follicles *in vitro* depends on the maintenance of oocyte-somite interactions, which can be well supported by tissue engineering applications. Using biological matrix such as alginate to encase human antral follicles can support their structure and promote their growth *in vitro* (Xu et al., 2009; Shea et al., 2014; Yin et al., 2016; Jones and Shikanov, 2019). Alginate and other biological materials provide an extracellular matrix-like substance for follicles. At the same time, they have good toughness and rigidity, which can satisfy the growth and expansion of follicles while maintaining the integrity of follicle units. At the same time, the follicle and the outer medium can exchange molecules (Skory et al., 2015). Based on the physiological process that displaces follicles from the cortex to the proximal medulla during development, it has also been proposed that biomechanical features and the physical environment within the ovary affect follicle development, with follicles located in the ovarian cortex is less likely to grow due to high collagen density, while follicles located in the medulla are exposed to easier growth due to a biomechanical environment that promotes hormonal responses (Woodruff and Shea, 2011). A team showed that alginate-encapsulated follicles could reach 110 μm in diameter in culture after 30 days, but the oocytes in these follicles could not reach MII stage (Xu et al., 2009), after which the team released some of antral follicles from the alginate hydrogel and then continued to culture them in low attachment plates for 40 days. The results showed that follicles cultured with alginate only produced oocytes that either remained at the germinal vesicle (GV) stage or degenerated, whereas 20% (5/20) of the follicles cultured using the two-step strategy reached the MII stage (Xiao et al., 2015). Studies using microfluidic techniques to wrap follicles have also demonstrated the role of mechanical heterogeneity in follicle development and ovulation (Choi et al., 2014). The finding that follicle function was influenced by the physical environment of three-dimensional (3D) follicle culture when hormonal stimulation was maintained (Xu et al., 2006; West et al., 2007; West-Farrell et al., 2009) further emphasized the importance of 3D culture for the isolated follicle culture environment. A range of 3D scaffolds has been developed to support follicle growth *in vitro*, including decellularized ovarian tissue (Laronda et al., 2015; Pors et al., 2019) and 3D microporous scaffolds (Laronda et al., 2017; Liverani et al., 2019). 3D scaffolds have great potential to provide a versatile option for engineering artificial ovarian creation due to their producibility (Dolmans and Amorim, 2019; Liverani et al., 2019; Dolmans et al., 2021). The most successful current culture systems that could support the full development of mouse oocytes from the primordial oocyte stage focused exclusively on the integrity of the oocyte-granulosa cell complexes (OGCs) and promote oocyte development (O'Brien et al., 2003) without attempting to grow intact antral follicles to the preovulatory stage *in vitro*. Isolated follicles cultured alone formed antral cavities within 6–8 days, followed by the release of the OGCs therein by slight squeezing of the follicle. Complexes with complete cumulus and adherent mural granulosa cells were then selected for further growth on membranes. After this step, oocytes of at least 100 mm with meiosis potential could be obtained (McLaughlin et al., 2018). The study removed oocyte complexes from surrounding cells and, further, may include transplants in women with leukemia, where IVG fertilization has previously been associated with continued transmission of cancerous cells.

Oocyte-somite interactions can also be enhanced by additive interventions (Li and Albertini, 2013). At the IVG stage, the addition of activin and low-dose FSH to IVA medium helps maintain intercellular connections, improves oocyte quality, and promotes follicle health and the formation of antrum, as demonstrated in experiments in dogs, cattle, and humans (Abir et al., 1997; Telfer et al., 2008; McLaughlin et al., 2010; McLaughlin et al., 2018). The addition of 200 ng/mL bFGF to IVG medium also promotes early follicle development in the human ovary (Wang et al., 2014). Supporting substrates or scaffolds may be beneficial, but they are not absolutely necessary to promote isolated follicle development, and the complex hormonal regulation of combined follicle growth suggests that the *in vitro* growth and development of human follicles need to address multiple challenges. More studies are needed in the future to compare different culture environments to find out which environment is most conducive to *in vitro* follicle development. Shown in Table 2.

**TABLE 2:** Additives in human follicle in IVG stage.

Additives	Dose	Mechanism	Follicles growth rate	MII rate	Author
FSH	1.5U/mL	Increase E2 production	62%	8%	McLaughlin M et al. (McLaughlin et al., 2018)
	1 ng/mL		42.7%	11%	R.A. Anderson et al. (Anderson et al., 2014)
	100 ng/mL		69.7%	—	McLaughlin et al. (McLaughlin et al., 2010)
	50 ng/mL		71.1%		
Actin-a	100 ng/mL	Regulate granulosa cell growth and differentiation	62%	8%	McLaughlin M et al. (McLaughlin et al., 2018)
			58%	30%	Evelyn E et al. (Telfer et al., 2008)
			69.7%; 71.1%	—	McLaughlin et al. (McLaughlin et al., 2010)
Ascorbic acid	50 μg/mL	Antioxidant that inhibits oocyte mitochondrial damage	62%	8%	McLaughlin M et al. (McLaughlin et al., 2018)
			42.7%	11%	R.A. Anderson et al. (Anderson et al., 2014)
			58%	30%	Evelyn E et al. (Telfer et al., 2008)
bFGF	200 ng/mL	Promote GC proliferation, suppress apoptosis in preantral follicles, and enhance early follicle cell differentiation	60%	30%	Tian-ren Wang (Wang et al., 2014)

### 2.3 Follicle *in vitro* maturation (IVM)

The IVM method for immature human oocytes has been in development for over 50 years (Edwards et al., 1969), but the first live birth after IVM was not reported until 1991 (Cha et al., 1991). IVM success is closely related to the oocyte source, with immature oocytes having a lower rate of successful maturation than oocytes harvested from stimulated ovaries (Nogueira et al., 2012; Chian et al., 2013). Oocytes generated during IVG must transfer to IVM medium for final meiosis and maturation. Most studies focus only on the maturation stage of oocytes, so the oocytes used in experiments are mainly from the COH cycle, while the maturation of IVG-derived oocytes has been less reported, but a review of these literature results is still helpful to optimize IVM after IVG. It was previously thought that oocyte diameter was critical for maintaining nuclear maturation and the ability to perform MII. In 2015, a study comparing alginate encapsulated culture with a two-step culture of separating follicles showed no significant difference in the diameter of oocytes that reached the MII stage and those that remained in the GV stage. This suggests that meiosis can be achieved in oocytes, although the size of the final follicle cannot reach the size of the preovulatory follicle *in vivo* (Xiao et al., 2015). In a later modified multi-step IVC system, follicular cavity formation was observed within 6–8 days after isolation of follicle culture and without waiting for follicle diameter to increase to pre-ovulation follicle level when COCs were removed for follow-up culture. Oocytes with diameters greater than 100 μm were obtained after IVM, and few oocytes developed to MII stage (McLaughlin et al., 2018). Alain Goughon (Gougeon, 1996) previously observed a 15%–24% atresia rate in human follicles with diameters between 0.9 and 2mm, and a 55%–77% atresia rate in follicles with diameters between 2 and 8 mm. The expression of FSH receptors on follicles in small sinuses was significantly higher than that in late follicular development (Jeppesen et al., 2013; Kristensen et al., 2018). The response to FSH stimulation may be enhanced and they may be more susceptible to stimulation and less prone to atresia. These results suggest that the IVC process can be implemented more directly and efficiently based on oocyte development, rather than follicle diameter.

At the end of the IVG process, IVM is performed on the harvested oocyte-cumulus complex to support meiosis recovery to the MII site. More recently, promising progress has been made in refining and optimizing IVM regimens through the use of a biphasic system (oocytes pretreated with C-type natriuretic peptide followed by routine IVM), with recent results showing significant improvements in maturation and oocyte quality (Sanchez et al., 2019). cAMP plays a crucial role in regulating oocyte maturation. The granulosa cells in the outer layer of the follicle contain C-type natriuretic peptide (CNP), while the surface natriuretic peptide receptor (NPR2) in cumulus cells around the oocyte. And the oocyte secretes paracrine factors that can promote the activation of NPR2 in cumulus cells. CNP of granulosa cells combines with activated NPR2 to produce cyclic guanosine monophosphate (cGMP) in oocytes and inhibit the activity of phosphodiesterase (PDE3A) in oocytes. Thus, high levels of cAMP in oocytes are maintained, leading to meiotic arrest of oocytes. When the LH level rises in the blood circulation, LH activates PDE3A and then downregulates the cAMP level in oocytes, which can restore the meiosis process of immature oocytes. Subsequently, oocytes reach the MII stage and fully mature (Grzesk and Nowaczyk, 2021). Therefore, the combination therapy of silotamide (PDE3-specific inhibitor) and capillarin (adenylate cyclase activator) can control the cAMP level in the cumulus complex, synchronize the maturation process of oocyte core and cytoplasm, and improve oocyte development and subsequent embryo development (Nogueira et al., 2006; Roy et al., 2021). The application of CNP in the culture medium can improve the success rate of meiosis of oocyte IVM (Sanchez et al., 2017). In addition to cAMP level, some other factors have also been proved that can promote oocyte maturation or benefit the subsequent embryonic development process: The addition of GDF9 can significantly increase the survival rate of fertilization and blastocysts, but does not affect the maturation rate of oocytes (Chatroudi et al., 2019). Growth hormone (GH) can increase the IVM maturation rate of human oocytes (Li et al., 2019). Melatonin has no apparent effect on the maturation rate of oocytes, but it can increase the blastocyst formation rate from 24.5% to 49.3% after fertilization by protecting mitochondria (Zou et al., 2020).

Based on the current exploration of the follicular multistage IVC system, it seems promising to obtain progeny by culturing human immature follicles *in vitro*. However, the current IVC system of human follicles is still limited by low MII rate, stability of fertilization ability and low safety. Thus, future research can be optimized for the three parts of the IVC system: (1)IVA: Clarifying the safe concentration and safe exposure time of existing activators to further explore the effective way of non-toxic activation of primordial follicles; (2)IVG: Exploring the best method and timing for separating follicle cells from the cortex, and developing a 3D follicle culture system to simulate the follicle development environment *in vivo* by combining microfluidics and other tissue engineering technologies; (3)IVM: Establishing a whole-process culture system to improve the maturation rate, fertilization ability and subsequent embryo development potential of IVG-derived oocytes. In conclusion, It is necessary to conduct a large number of studies to optimize the various stages of follicle development *in vitro* and establish a relatively unified, standardized, efficient and safe culture system before applying multistage IVC technology to the clinical practice. Shown in Table 3.

**TABLE 3:** Additives in human follicle in IVM stage.

Additives	Mechanism/Pathway	Effect	Author
Silotamide and capillarin	Silotamide (PDE3-specific inhibitor) and capillarin (adenylate cyclase activator)	Control the cAMP level in the cumulus complex, synchronize the maturation process of oocyte core and cytoplasm, and improve oocyte development and subsequent embryo development	Nogueira, D. et al. (Nogueira et al., 2006)
			Roy, P. K. et al. (Roy et al., 2021)
GDF-9	Induce the mitotic division of granulosa cells to suppress the FSH receptor expression those cells; Induce the expression of kit ligand mRNA in granulosa cells	Increase the survival rate of fertilization and blastocysts, but has no effect on the maturation rate of oocytes	Chatroudi, M. H. et al. (Chatroudi et al., 2019)
Growth hormone	Accelerate meiotic progression	Increase the IVM maturation rate of human oocytes	Li, Y. et al. (Li et al., 2019)
Melatonin	A potent antioxidant	has no apparent effect on the maturation rate of oocytes, but it can increase the blastocyst formation rate from 24.5% to 49.3% after fertilization by protecting mitochondria	Zou, H. et al. (Zou et al., 2020)

## 3 Oogenesis, growth, and maturation induced by pluripotent stem cells (PSCs)

Utilizing pluripotent stem cells (PSCs) to induce oocytes has emerged as a new method of fertility preservation with the introduction of induced pluripotent stem cell (iPSC) technology in 2006. Since the oocytes of mice and human originate differently, mouse PSCs (mPSCs) and human PSCs (hPSCs) have slightly different induction techniques.

### 3.1 *In vitro* induction of mouse female oocytes by mouse PSCs

The researchers discovered that mPSCs demonstrate more potent germ cell capacity in 2iLIF medium including inhibitors of LIF and MAPK/GSK3 pathway. The subsequent induction of mPSCs in 2iLIF media including ActA, bFGF, and GMEM/KSR for 2 days resulted in the formation of ectoderm epiblast-like cells (EpiLCs). EpiLCs are appropriate precursors for producing mouse primordial germ cell like-cells (PGCLC), as they have gaenteral ectoderm cellular features (Sarma et al., 2019). About 30% of EpiLCs were transformed to PGCLCs after subsequent induction for 4–6 days under BMP4, LIF, SCF, and EGF conditions (Hayashi et al., 2011). These mouse PGCLCs displayed comparable transcriptome and epigenetic profiles to mouse PGCS, and the process of their production underwent dynamic epigenetic regulation similar to that of PGC differentiation. However, it is more challenging to promote mouse PGCLCs undergoing further differentiation. Previously, “reconstituted ovaries” were created by aggregating mouse PGCLCs with mouse female gonadal somatic cells, undergo X-reactivation, imprint erasure, and cyst formation, and exhibit meiotic potential and then transplanted under mouse ovarian bursa (Hayashi et al., 2012). Upon transplantation, mouse PGCLCs in the reconstituted ovaries mature into germinal vesicle-stage oocytes (Hayashi et al., 2012). These oocytes can develop into MII oocytes following IVM and obtain healthy fertile offspring after IVF fertilization (Hayashi et al., 2012). However, oogenesis in this method is not completely done *in vitro*. In subsequent studies, esearchers have attempted to rebuild mammalian oogenesis entirely from mouse PGC *in vitro* using an estrogen-receptor antagonist that promotes normal follicle formation, which in turn is crucial for supporting oocyte growth (Morohaku et al., 2016). The fundamental events in oogenesis (i.e., meiosis, oocyte growth, and genomic imprinting) were then reproduced in the culture system. The IVA, IVG, and IVM processes are continued after *in vitro* differentiation (IVD), and the ultimately generated MII oocytes can result in healthy and fertile offspring through IVF (Morohaku et al., 2016). Taking the above breakthroughs into account, it is not difficult to connect the entire process of *in vitro* induction of mouse PSC into mouse oocytes by combining the induction of mouse PGCLC and *in vitro* oogenesis of PGC. Namely, mouse PSCs were initially differentiated into mouse EpiLCs, and then mouse PGCLCs were produced under the influence of BMP4, LIF, SCF, and EGF. Via IVD, IVA, IVG, and IVM procedures, the MII oocytes were gradually produced, undergo fertilization to produce healthy, viable offspring which show normal levels of body weight, survival rate, fertility, and gene expression (Hikabe et al., 2016).

According to the landmark study above, “reconstituted ovaries” containing female gonadal somatic cells played a vital role in promoting further oocyte differentiation (Hayashi et al., 2012; Hikabe et al., 2016). However, to clarify the unclear mechanism of oocyte differentiation, it is crucial to investigate how to induce oocytes without ovarian somatic cells. Overexpression of the pluripotency gene NANOG individually could promote the formation of PGC. NANOG is upstream of PRDM14 and BLIMP1, and NANOG binds to their enhancers (Murakami et al., 2016). Germline induction can be facilitated by the overexpression of PRDM14 alone or by the combined expression of the three germline genes BLIMP1, PRDM14, and TFAP2C (Nakaki et al., 2013). Transient overexpression of DAZL, which is required for the differentiation and growth of germ cells, can inhibit the expression of NANOG and promote the formation and meiosis progression of oocyte-like cells (Yu et al., 2009). Additionally, a cluster of transcription factors, including Nobox, Figla, Tbpl2, Sohlh1, Stat3, Dynll1, Sub1, and Lhx8 (Hamazaki et al., 2020), can encourage the development of primordial follicles into primary follicles. The primary follicles are placed in the reconstituted ovarian environment after being cultivated. Even though there was no distinct epigenetic reprogramming or meiosis process at the end, the fact that the procured oocyte-like cells could mature, fertilize, divide, and progress to the 8-cell stage also demonstrated the independence of mouse oocyte maturation, epigenetic reprogramming, and meiosis. PGCLCs can be amplified by the enhanced cAMP expression (Ohta et al., 2017). BMP and RA can commence initiation differentiation without the involvement of ovarian somatic cells since BMP2 and RA cooperate to further induce PGCLCs to differentiate into primitive oocyst-like cells that express VASA and SCP3 (Ohta et al., 2017). The discovery of the cytokines and transcription factors mentioned above provides a basis for encouraging oocyte differentiation and starting meiosis.

In conclusion, researchers have been able to induce MII oocytes from mouse PSC and obtain healthy and fertile offspring after 2 decades of effort. Both the technique of overexpressing the relevant genes by regulating the genetic level and the strategy of constructing an ovarian-like microenvironment using ovarian somatic cells were successful in generating mouse female GCs from mouse PSC. Therefore, the most effective method for inducing female GCs in mice has been recognized as the production of PGCLCs from PSCs by EpiLCs and subsequent binding of PGCLCs to mouse female gonadal somatic cells (Irie et al., 2018; Hayashi, 2019). The mechanism of female GCs can be revealed through further induction scheme optimization, which can increase the culture system’s induction efficiency.

### 3.2 *In vitro* induction of human female oocytes by human PSCs

Treated with 4i medium (an inhibitor of MAPK, GSK3, p38, and JNK), hPSCs can be brought back to the naive stage (Gafni et al., 2013), at which hPSCs at the naive state can be exposed to TGFβ, βFGF, and LIF for 2 days in a similar method to the induction of mouse PGCLC. Then, after 8 days of induction by BMP2/4, LIF, SCF, and EGF (Irie et al., 2015), it is possible to differentiate into human PGCLCs (hPGCLCs). In a different study, hiPSCs were grown with FGF and a free-feeding layer, then stimulated for 2 days with ActA and the WNT signaling agonist (CHIR99021). The acquired cells displayed increased pluripotency and expressed mesodermal genes that corresponded to incipient mesoderm-like cells (iMeLCs) Then, for a further 4 days, it was cultured in GMEM/KSR, BMP4, LIF, SCF, and EGF to produce hPGCLCs that corresponded to the seventh week of embryonic development (Sasaki et al., 2015) In addition, Another team has accomplished the differentiation of hPSCs into hPGCLCs in a concentration-dependent manner. ActA, FGF, and low concentration (5 ng/mL) BMP4 were first utilized to convert hPSCs into mesodermal cells, and subsequently high concentration (100 ng/mL) BMP4 was used to stimulate the differentiation of mesodermal cells into hPGCLCs (Sugawa et al., 2015). The mechanism of female GCs differentiation was increasingly clarified by successful *in vitro* reconstruction of hPGCLCs, which promoted the induction of hPGCLCs differentiation in more efficient ways.

As for mice, the coculture of mouse PGCLCs with mouse female gonadal somatic cells can be applied to the next step of induction. But obtaining human female gonadal somatic cells is challenging. Therefore, it is necessary to explore an induction strategy without the coculture of human female gonadal somatic cells. According to Jung et al., overexpressing DAZL and BOULE can lead hESC to leave the pluripotent state and enter meiosis. GDF9 and BMP15 can then be added to speed up the induction of FLCs, which can subsequently express ZP2 and NOBOX and generate estradiol after implantation in the renal sac of the mouse. Currently, ovarian somatic cells are necessary for initiating oocyte differentiation due to the interaction between oocytes and somatic cells. In a recent study, researchers swapped over human embryonic ovarian cells with mouse ovarian cells and then incubated the resulting “xenorecombinant ovary” for 121 days (Yamashiro et al., 2018). The early PGC genes BLIMP1, TFAP2C, SOX17, and NANOS3 were discovered to undergo downregulation upon induction. The critical meiosis genes DMC1, H2AX, or SCP1 were not adequately upregulated during induction, while DAZL, VASA, and RA response genes (STR8 and SCP3) were. The cells inducing in “xenogeneic recombination ovary” correspond to oocytes and have DNA demethylation and imprint erasability similar to those of embryos at 10 weeks of development *in vivo*. It indicates that the cells are in the process of entering meiosis but have not yet done so, which might be a consequence of inadequate meiotic signaling in xenogeneic mouse ovarian cells. Theoretically, human ovarian somatic cells induced by hPSCs may be applied in the induction of hPGCLCs. By transplanting hipScS-induced granulosa cells into POF mice ovaries, previous research has demonstrated that these cells can secrete hormones that improve POF and promote follicle formation (Liu et al., 2016; Lipskind et al., 2018). Additionally, one of the future study objectives might be to determine whether hipScS-derived granulosa cells can be employed as substitutes for human ovarian somatic cells and combine with PGCLCs to generate recombinant ovaries to further accelerate oocyte differentiation and production.

Up to now, human oogonium-like cells have been successfully induced by constructing hiPSCs and mouse female gonadal somatic cells in “xenogenic recombinant ovary” (Yamashiro et al., 2018). These efforts and advancements offer opportunities for additional research into the genes specific to female GCs, PGCs migratory pathways, sexual differentiation, and initiation of meiosis, even if there is still a long way to go before oocytes can be utilized clinically to create fertile babies. Besides developing effective and securing protocols for the differentiation of PGCLC into genetically and epigenetically normal, patient-specific oocytes, it is now more important to identify the critical molecules that facilitate the maturation of hPGCLCs and to understand the mechanisms of the ovarian microenvironment.

Recently, genome-wide DNA methylation maps during human preimplantation development were revealed by global scale landscape sequencing of single-cell chromatin in human preimplantation embryos (Li et al., 2018), offering us some information on how humans PGC differentiation before the second week of embryonic development. Furthermore, female GCs differentiation was split into three stages, including the RA response stage, premeiotic stage, and primordial follicle stage, using single-cell transcriptome and epigenome sequencing technology (Wen and Tang, 2019). Each stage corresponded to a distinctive level of gene expression and epigenetic regulation. Single-cell methods for genome-wide DNA methylation and chromatin analysis can be employed to investigate the epigenetic regulation network of female GC at various developmental stages (Wang et al., 2019). Likewise, methods to improve the cultural environment are being researched. Recently, a redesigned approach that combines a low adhesion cell culture plate with a 3D induction system based on methylcellulose has made it feasible to induce PGCLC on a massive scale to boost production efficiency (Wang et al., 2019). The 3D artificial ovary mentioned above, except for the 3D induction system, can facilitate oocyte development. Future research will examine whether a 3D artificial ovary can provide a condition for PSC induction that is more comparable to the ovarian microenvironment *in vivo*. Ultimately, ethical issues in reproductive medicine have been receiving attention from both the scientific community and the public. Especially concerning the ethical issues expressed by the induction of GCs in human females, questions regarding stem cell origin, technical safety, clinical application of generated cells, and epigenetic regulation of offspring remain common.

Although hPSCs have not yet been properly converted into human MII oocytes, it is theoretically feasible to combine the already used approaches to produce MII oocytes *in vitro* through hPGCLCs induction, iPSC to granulosa cell induction, IVD, IVA, IVG and IVM. If the idea was successful, this will result in a breakthrough in our comprehension of the complicated biological processes involved in oocyte development, an innovative cell model for testing sterility-related drugs, and fresh perspectives on ovarian disease research and the preservation of future fertility in OC and POF patients.

## 4 Artificial ovary construction technology

The artificial ovaries focus on constructing biological material used to construct a stromal environment in which follicles can proliferate and ensure sex hormone secretion. This biological material should be 1) biosafety and tolerable by the human body, 2) high-temperature resistant due to the human body temperature (Chen et al., 2022), 3) liable for cell adhesion, proliferation, and differentiation, and 4) allow the dissemination of nutrients, growth factors, and oxygen. The final goal of artificial ovaries is to be retransplanted into the human body, so the tolerability and biosafety of its components are very important. Since human follicles vary greatly in diameter during growth (from 19 to 30 μm to 100–110 μm), the material should be degradable and conducive to follicle growth and migration. Furthermore, given the close signaling exchange between the follicle and the endofollicular and intrafollicular environment, this material should be highly permeable to allow diffusion of nutrients and signaling in and out of it. A number of tissue engineering materials suitable for artificial ovaries have been developed, ranging from natural materials (collagen, plasma clots, alginate, fibrin, decellularized tissue, *etc.*) to synthetic polymers (polyethylene glycol, 3D printed ovaries, *etc.*) as shown in Table 4, with encouraging results in animal research models. Laronda et al. isolated follicles from cryopreserved human ovarian tissues to form an artificial ovary and transplanted them into ovariectomized adult mice,6 out of 7 ovariectomized mice with artificial ovary implanted had recovered hormone cycle in 4 weeks (Laronda et al., 2015). Kniazeva et al. extracted follicles from young female mice and encapsulated them with artificial ovaries, mice for subsequent transplantation, 33% of female mice deliver offspring (Kniazeva et al., 2015). Natural polymers are usually less rigid but have advantages in cell adhesion, migration and signal communication. Synthetic polymers have better mechanical properties to support human transplantation but are not conducive to nutrient exchange and signal crosstalk (Reddy et al., 2021), and current research is directed at combining the two to better prepare artificial ovarian materials.

**TABLE 4:** Meterials applied in the artificial ovary construction technology.

Materials	Advantages	Disadvantages	Author	Specie	Year	Outcomes
Collagen	Plasma clots allow follicles to progress to the sinus stage	The plasma clot’s poor composition and quick degradation lead to greater follicle loss	Telfe et al. (Telfer et al., 1990)	Mice	1990	The follicles could develop and mature 5 days after transplantation, and mature follicles from this grafted gel could eventually form embryos by IVF.
Plasma clots			Gosden et al. (Gosden, 1990)	Mice	1990	11 of 15 mice became pregnant and 2 produced offspring
Plasma clots			Dolmans et al. (Dolmans et al., 2007; Dolmans et al., 2008)	Human	2007	Five months after transplantation, stage II and antral follicles could be found in the clot, but the plasma clot degraded rapidly, resulting in the loss of a large number of follicles
Alginate	The rigidity prevents structural degradation	Human follicles lack alginate lyase, which would limit angiogenesis and further follicle	Rios et al. (Rios et al., 2018)	Mice	2018	Many follicles could develop into antral follicles, and mature follicles could be successfully fertilized by ICSI.
			Wang, T. R. et al. (Wang et al., 2014)	Human	2014	Human primordial follicles encapsulated in alginate gel and cultured *in vitro* for 8 days can develop, and some of them can reach the preantral stage
Fibrin	High bioadhesive properties and low inflammation after transplantation into humans	high rate of degradation and after degradation follicles lose structural support	Paulini et al. (Paulini et al., 2016)	Human	2016	Many follicles could grow to secondary follicles after 7 days
			Smith, R. M. *et al* (Smith et al., 2014)	Mice	2014	Isolated primary mouse follicles could also develop to the antral stage and hormone production can be detected
Fibrin-alginate	It has good degradability and rigidity, maintains the connection communication between the internal cells and follicle structure	—	Shikanov, A. et al. (Shikanov et al., 2009)	Mice	2009	Short-term culture of mice secondly follicle encapsulated with an interpenetrating network composed of fibrin-alginate resulted in higher oocyte MII rates than culture with alginate or fibrin alone
			Brito, I. R. et al. (Brito et al., 2016)	Goat	2016	The maturation rate of isolated caprine follicles cultured in a fibrin-alginate matrix for a longer period of time (30 days) was higher than that cultured with alginate alone
The decellularized ovarian extracellular matrix	Highly mimic the natural ovary *in vivo*, allowing for cell adhesion and growth	Xenogeneic scaffolds may induce a high risk of immune response and may also induce certain diseases, such as viruses or cells residues from the donor	Laronda et al. (Laronda et al., 2015)	Mouse follicles and decellularized bovine ovarian scaffolds	2015	After 2 weeks of transplantation, an antral follicle could be found in the transplanted scaffold
			Hassanpour et al. (Hassanpour et al., 2018)	Rat follicles and decellularized human ovary	2018	Four weeks after surgery, hormones and primordial or primary follicle-like structures were detected in this suitable cytocompatibility scaffold
			Pors et al. (Pors et al., 2019)	Human follicles and decellularized human scaffold	2019	They successfully implanted isolated human follicles in a decellularized human scaffold and reimplanted them in rats for 3 weeks
Polyethylene glycol (PEG)	Synthetic polymers can be tailored to the different stiffness of natural ovaries to meet different clinical requirements	Degradation of synthetic polymers is toxic and the degradation products tend to cause immune reactions	Kim et al. (Kim et al., 2016)	Mouse	2016	All stages of follicles and corpora lutea could be found in the scaffold 30 days after transplantation
Bio-3D printing	Bio-3D printing allows precise adjustment of the pore diameter and thickness of the scaffold, and also controls properties such as rigidity of the scaffold to meet clinical needs	—	laronda et al. (Laronda et al., 2017)	Mice	2020	Mature follicles were found 3 weeks after implantation, and after 10 weeks, these transplanted mice could be mated to produce normal offspring
			Raffel, N. et al. (Raffel et al., 2019)	Pig	2019	After 10 days of *in vitro* culture, the follicles adhered well to the scaffold, developed well, and had a high survival rate
			Yoon, H. J. et al. (Yoon et al., 2021)	Rat	2021	The release of hormones that significantly aid the restoration of endocrine function can completely regenerate the endometrium

Collagen and plasma clots were the first natural 3D scaffolding materials used for isolated primordial follicle encapsulation. In 1990, Telfe et al. cultured isolated primary mouse follicles in collagen for 5 days and then transplanted them into ovariectomized mice; the follicles could develop and mature 5 days after transplantation, and mature follicles from this grafted gel could eventually form embryos by IVF, while sufficient hormones were produced within the grafted gel to support the vaginal opening and keratinization of the vaginal epithelium, and angiogenesis appeared in the gel as well (Telfer et al., 1990). Gosden et al. isolated primordial follicles from mice, cultured them in plasma clots, and then transplanted them back into ovariectomized mice. 11 of 15 mice became pregnant and 2 produced offspring (Gosden, 1990). Dolmans et al. isolated human primordial follicles, encapsulated them in plasma clots, and xenotransplanted them into immunodeficient mice. Five months after transplantation, stage II and antral follicles could be found in the clot, but the plasma clot degraded rapidly, resulting in the loss of a large number of follicles (Dolmans et al., 2007; Dolmans et al., 2008). Overall, plasma clots allow follicles to progress to the sinus stage, however the plasma clot’s poor composition and quick degradation lead to greater follicle loss (Dolmans et al., 2007; Dolmans et al., 2008).

To address the problem of degradation, researchers developed alginate, a polysaccharide-based natural polymer derived from algae whose rigidity prevents structural degradation. Rios et al. encapsulated mouse isolated follicles into an alginate matrix and transplanted them back into ovariectomized mice. Many follicles could develop into antral follicles, and mature follicles could be successfully fertilized by intracytoplasmic sperm injection (ICSI) (Rios et al., 2018). It has been reported that isolated human primordial follicles encapsulated in alginate gel and cultured *in vitro* for 8 days can develop, and some of them can reach the preantral stage (Wang et al., 2014). However, when the culture *in vitro* last for a longer period of time (about 30 days), follicles grew to the antral stage, but many follicles degenerated and stopped growing after further incubation (Yin et al., 2016). The reason for this may be that human follicles are 2-fold larger than mice, and the lack of alginate lyase, which prevents degradation of alginate, would limit angiogenesis and further follicle (Chiti et al., 2016).

Fibrin is an alternative natural polymer to plasma colt with high bioadhesive properties and low inflammation after transplantation into humans, and has been widely used in tissue engineering. Paulini et al. encapsulated isolated human primordial follicles in fibrin gel and transplanted them into mice, and many follicles could grow to secondary follicles after 7 days (Paulini et al., 2016). Isolated primary mouse follicles cultured in fibronectin gels for long periods (21 days) could also develop to the antral stage and hormone production can be detected in mice (Smith et al., 2014). However, fibrin has the same high rate of degradation as plasma clots and collagen in humans, and after degradation follicles lose structural support due to inherent inhibitors such as fibrinolytic enzymes in humans. Follicles migrate and grow in different ovarian structures due to the different cortices and medullae of the natural ovary (Laronda, 2020). Short-term culture of mice secondly follicle encapsulated with an interpenetrating network composed of fibrin-alginate resulted in higher oocyte MII rates than culture with alginate or fibrin alone (Shikanov et al., 2009). The maturation rate of isolated caprine follicles cultured in a fibrin-alginate matrix for a longer period (30 days) was higher than that cultured with alginate alone (Brito et al., 2016). Thus, the fibrin-alginate matrix combination has better degradability and rigidity, which facilitates follicle survival and proliferation. Moreover, to build a synthetic ovarian scaffold, slowly degrading alginate and fibrin can combine their benefits while maintaining the connection communication between the internal cells and follicle structure (Zhou et al., 2015).

The decellularized ovarian extracellular matrix is another natural matrix obtained by removing the cellular components of the natural ovary and can highly mimic the natural ovary *in vivo*, allowing for cell adhesion and growth. Decellularized tissues have been applied in the liver (Fares et al., 2021), lung (Dabaghi et al., 2021), and heart (Ercan et al., 2021). Laronda et al. implanted isolated mouse follicles into decellularized bovine ovarian scaffolds and transplanted them into immunocompetent normal ovariectomized mice. After 2 weeks of transplantation, an antral follicle could be found in the transplanted scaffold (Laronda et al., 2015). Hassanpour et al. decellularized human ovary and encapsulated it with isolated rat follicles, then reimplanted it in rats. Four weeks after surgery, hormones and primordial or primary follicle-like structures were detected in this suitable cytocompatibility scaffold (Hassanpour et al., 2018). Pors et al. also successfully implanted isolated human follicles in a decellularized human scaffold and reimplanted them in rats for 3 weeks (Pors et al., 2019). However, xenogeneic scaffolds may induce a high risk of immune response and may also induce certain diseases, such as viruses or cells residues from the donor (Massaro et al., 2021).

In contrast to natural polymers, synthetic polymers can be tailored to the different stiffness of natural ovaries to meet different clinical requirements (Dolmans and Amorim, 2019). Polyethylene glycol (PEG) is widely used in engineering, and oxygen and carbon are the main components of PEG. Kim et al. used PEG hydrogels to encapsulate isolated mouse follicles and transplant them into ovariectomized mice, and all stages of follicles and corpora lutea could be found in the scaffold 30 days after transplantation. After 60 days of transplantation, hormone levels were significantly increased and functional vessels could also be detected in the scaffold (Kim et al., 2016). In other studies, PEG-superoxide dismutase, which promotes follicle growth, has been combined with polytetrafluoroethylene membrane, which successfully prevents graft immune recognition and restores endocrine function in mice with ovariectomies (David et al., 2017). However, degradation of synthetic polymers is toxic and the degradation products tend to cause immune reactions (Shiraishi and Yokoyama, 2019).

Bio-3D printing allows precise adjustment of the pore diameter and thickness of the scaffold, and also controls properties such as rigidity of the scaffold to meet clinical needs. It can fabricate scaffolds layer by layer to produce tissue-mimicking structures (Zubizarreta and Xiao, 2020). Laronda et al. used gelatin as3D ink to print 3D scaffold crosslink with a support diameter of 250 μm and a pore diameter of 350 μm. After implanting isolated mouse follicles into 3D printed scaffolds, the scaffolds were transplanted into ovariectomized mice for 7 days after implantation without the addition of exogenous angiogenic factors. Mature follicles were found 3 weeks after implantation, and after 10 weeks, these transplanted mice could be mated to produce normal offspring (Laronda et al., 2017). It has also been reported that isolated porcine follicles were implanted in gelatin and poly (epsilon-caprolactone) (PCL)-printed scaffolds to construct scaffold structures with a pore size of 300 μm and a scaffold diameter of 1 μm. After 10 days of *in vitro* culture, the follicles adhered well to the scaffold, developed well, and had a high survival rate (Raffel et al., 2019). In terms of the crucial limitations of how vascularization can be achieved in the artificial ovary, a recent research team used 3D printing technology to create numerous microvascular channels in hydrogels. They then coated rat oocytes layer by layer with autologous granulosa cells, follicular theca cells, basal basal-like ECM, gelatin, and hydrogels with microchannels, and implanted them into the ischemic hind limbs of ovaries removed rats. The release of hormones that significantly aid the restoration of endocrine function can completely regenerate the endometrium (Yoon et al., 2021).

Growth factors play an equally important role. Growth factors such as vascular endothelial growth factor (VEGF) and basic fibroblast growth factor (bFGF) promote artificial ovarian angiogenesis and reduce apoptosis *in vivo*. Shikanov et al. encapsulated ovarian tissue with VEGF in a fibrin gel and transplanted it back into bilateral oophorectomy mice. After 2 weeks of transplantation, the VEGF-containing gel survived two times (Shikanov et al., 2011) as many follicles and blood vessels as the control group (102). In another study, ovarian tissue was also wrapped in a fibrin gel along with bFGF and then transplanted under the skin of mice. Seven days after transplantation, the bFGF group had higher follicle survival and proliferation rates, lower rates of follicular and ovarian apoptosis, and higher rates of angiogenesis compared to the non-bFGF group (Gao et al., 2013). The apoptosis suppressor sphingosine-1-phosphate (S1P) is an apoptosis suppressor capable of inducing cell survival and proliferation. It is a signaling sphingolipid that can act as an intracellular second messenger and extracellular ligand for G protein-coupled receptors. It also regulates angiogenesis and vascular stability (Soleimani et al., 2011). Soleimani et al. reported that human ovaries were transplanted with S1P into severe combined immunodeficient (SCID) mice. Ten days after transplantation, the grafts showed significantly increased vascular density, angiogenesis, and ovarian cell proliferation, and lower follicular apoptosis compared to controls (Soleimani et al., 2011). Another study implanted follicles with S1P and VEGF in a fibrin scaffold and produced twice as many primordial follicles, vessels, and progeny as controls (Ladanyi et al., 2017). Besides, the addition of platelet-derived growth factor (PDGF), BMP4 (Felder et al., 2019), and other substances can help to further increase the vascularization of the graft and the restoration of follicular activity.

Although artificial ovary technology has made tremendous progress in mice, it is still in the preliminary stages of study in large animals and humans since their grafts are much larger than those of mice in terms of size, quantity, and size of follicles. Therefore, when graft scaffold gets larger, it is an urgent problem to be solved how to design vascular channels in the scaffold or advance the vascularization (Yoon et al., 2021) of the implant with the graft scaffold growing larger while maintaining follicle vitality. The ovary’s cortex and medulla have different functions, and the arrangement of the follicle and its constituent parts in space (Quan et al., 2020) will also have an impact on how well it performs. In order to maximize cell-cell interaction, enhance ovarian function, and lengthen the implant’s longevity, structure of ovary should be considered when building the ovarian organ.

## 5 Construction technology of ovarian organoids

Organoid technology is one of the most important breakthroughs in the area of tissue engineering research in the past decade and was rated as one of the top ten discoveries by 《Science》. It refers to the application of 3D culture technology to produce matrix glue as a growth scaffold, regulate a range of cell internal and external signals, and encourage the cells with stem cell potential to generate tissue structures resembling those derived from corresponding organs. Organoids, particularly tumor organoids, have the generally stable phenotypes and genetic properties and exhibit a high degree of histological similarity to real organs. The morphology and size of organoids among individuals remain largely uniform, in contrast to tumor cell lines and xenotransplantation models, maintaining the heterogeneity of the source tumor and the heterogeneity between patients. It offers a quick and excellent technical platform for the study of tumor pathogenesis, drug screening, personalized precision medicine, regenerative medicine, and other fields. At present, organogenesis has been reported in ovaries, fallopian tubes, endometrium, cervix and trophoblasts.

In terms of ovaries, Jung et al. (Jung et al., 2017) first established FLCs structures similar to follicular organoids by using HESCs in 2017. Four years later, Ji Wu et al. first used female germline stem cells (FGSCs) and a three-dimensional culture system to induce the generation of ovarian organoids. FGSCs were sorted by magnetic activated cell sorting (MACS) and cultured with α-MEM supplement with 10% fetal bovine serum, 10 ng/mL mouse leukemia inhibitory factor, 10 ng/mL mouse bFGF, 10 ng/mL EGF, 40 ng/mL mouse glial cell line-derived neurotrophic factor, 1 mM non-essential amino acids, 2 mM L-glutamine, 10 mg/mL penicillin, 30 mg/mL pyruvate and β-mercaptoethanol (Li et al., 2021). The ovarian organoids contained six kinds of ovarian cells, including germ cells, granulosa cells and theca cells. Oocytes could be produced and had endocrine functions. Using this model, normal mouse offspring could be produced. In addition, drug toxicity could be tested and it was found that ascorbic acid treatment had a beneficial effect on the maintenance of germ cell numbers, whereas salinomycin affects the formation of ovarian organoids and the maintenance of germ cell populations by inducing apoptosis (Li et al., 2021). Ovarian organoid model can play an important role in the study of oocyte development and screening of drugs promoting oocyte development *in vitro*. Using ovarian organoids, it was found that topologically dependent domains were stable during germ stem cell differentiation, but chromatin interactions changed in surprising ways, altering 35 percent of inactive and active chromosomal compartments throughout the genome (Luo et al., 2021). Recently, George Church et al. published a study on the directed differentiation of human iPSC cells into functional, fully human ovarian organoids, which can support oocyte maturation, follicle development and sex hormone secretion (Pierson Smela et al., 2023). The results showed that simultaneous overexpression of transcription factors NR5A1 and RUNX1/RUNX2 could induce the redifferentiation of iPSCs into granulosa cells. These granuloid cells had a transcriptome similar to that of human fetal ovarian cells. More importantly, these granuloid cells induced from human iPSCs could be co-cultured with hPGCLC also induced from human iPSCs to form ovarian organoids to aid oocyte development.

Currently, a two-step approach to bioengineering ovaries using ovarian organoids combined with artificial ovarian technology has been summarized: the first step aims to create 3D biological scaffolds, obtained mainly through whole-organ acellular technology, which can simulate the natural ovarian environment *in vitro*, allowing the maintenance of the original tissue microstructure and biological signals. The second step involves the isolation of purified FGSCs using MACS, which are capable of further differentiation when organoid techniques are used and can be used for the refilling of the ovarian acellular biological scaffold (Pennarossa et al., 2021). The combination of these two techniques provides a powerful tool for *in vitro* reconstruction of bioengineered ovaries, which may be a promising solution for restoring hormonal and reproductive function. However, the current problem is that the ability of organoids to generate oocytes *in vitro* is generally low, especially the low IVM rate (Luo et al., 2021), and it is necessary to establish a culture system that can not only effectively expand FGSCs but also maintain the uniformity of FGSCs differentiation into oocytes. Changing the culture substrate, such as water-soluble, Fzd subtype-specific “next-generation surrogate” (NGS) Wnts that hetero-dimerize Fzd and Lrp6 solves the problem of Wnt lipidation and Wnt-Frizzled (Fzd) cross-reactivity, supports the long-term expansion of many different types of organoids such as ovaries. This culture condition is considered to be superior to Wnt3a conditioned medium in organoid dilatation and single cell organoid growth (Miao et al., 2020). Recently, 3D microenvironments have been shown to trigger mitochondrial dysfunction during follicular growth *in vitro* (Takashima et al., 2021). Adding 100 nM MitoQ to the medium promotes follicular growth and maturation *in vitro* during organoid growth, while removing ROS, reducing oxidative damage, and restoring mitochondrial membrane potential in oocytes (Wang et al., 2023a). Inhibition of EED activity could promote the survival of FGSCs and significantly inhibit their apoptosis during *in vitro* differentiation. EED226 treatment and processing of FGSCs can enhance the expression of Oct4 and inhibit the expression of P53 and P63 by reducing the enrichment of H3K27me3 in Oct4 promoter and exon. Specifically improve the survival rate of FGSC (Wang et al., 2023b).

In addition to ovarian organoids, organoids of various pathological types of ovarian cancer have been reported, including epithelial ovarian cancers such as serous carcinoma, endometrioid carcinoma, clear cell carcinoma and mucous carcinoma (Kopper et al., 2019), as well as ovarian sarcoma (Phan et al., 2019) and even borderline ovarian tumor (Maru et al., 2019). The construction of ovarian organoids is shown in Table 5. Patient-derived ovarian cancer organoids can be successfully modeled in a short period and compare drug high-throughput e*xperimentations* in different patients, contributing to the accurate screening of anti-ovarian cancer drugs. Generally, the present cultured organoids own a simple construction, while their lack of immune cells, blood vessels, innervation, matrix, and vasculature distinguish them from real organs. Future studies will concentrate on how to create a tumor microenvironment in an *in vitro* culture system. The recently reported gas-liquid interface culture (Ye et al., 2020) and microfluidic technology (Li et al., 2017) will make it possible to co-culture organoids with immune cells and mesenchymal cells, advancing the study of organoids in immunotherapy. Besides, organoid development will be greatly accelerated by other innovations, such as refining the composition of culture medium, reducing the variety of culture conditions, using CRISPR/Cas9 gene editing technology, RNA-seq technology, bioprinting, and organoids-on-chips technology.

**TABLE 5:** Organoids derived from healthy and pathological ovary tissues.

Tissue source	Organoid medium	Specie	Application	References
Female germline stem cells (FGSCs) from 6- or 16-week-old mice	Three-dimensional culture medium was based on Glasgow MEM supplemented with 15% Knockout serum replacement, 1.5 μM retinoic acid, 2 mM L-glutamine, 1 mM non-essential amino acids, 2 mM L-glutamine, 30 mg/mL pyruvate, 100 mM β-mercaptoethanol, 20 ng/mL mEGF, 10 ng/mL bFGF, 10 ng/mL mouse glial cell line-derived neurotrophic factor, and 10 ng/mL mouse leukemia inhibitory factor, 30 mg/mL penicillin, and 75 mg/mL streptomycin. The α-MEM-based medium was α-MEM supplemented with 2% FBS, 2 mM L-glutamine, 100–300 μM ascorbic acid, 20 ng/mL mEGF, 50 mM β-mercaptoethanol, 30 mg/mL penicillin and 75 mg/mL streptomycin. StemPro-34-based medium was StemPro-34 SFM supplemented with 10% FBS, 2 mM L-glutamine, 100–300 μM ascorbic acid, 20 ng/mL mEGF, 50 mM β-mercaptoethanol, 30 mg/mL penicillin, and 75 mg/mL streptomycin, and 800 nM ICI182780 was used depending on the experimental context	Mice	Production of oocytes and offspring; Secretion of hormones; drug toxicological detection	Li et al. (2021)
Spermatogonial stem cells (SSCs) were isolated from Testes of 6-day-old pou5f1-GFP transgenic mice or pou5f1/GFP transgenic mice × C57BL/6 F1 hybrid mice	GMEM supplemented with 15% Knockout serum replacement, 1.5 μM retinoic acid, 2 mM L-glutamine, 1 mM non-essential amino acids, 2 mM L-glutamine, 30 mg/mL pyruvate, 100 mM β-mercaptoethanol, 30 mg/L penicillin, and 75 mg/L streptomycin.The α-MEM supplemented with 2% FBS, 2 mM L-glutamine, 200 μM ascorbic acid, 50 mM β-mercaptoethanol, 30 mg/lpenicillin, and 75 mg/L streptomycin. The StemPro-34 SFM supplemented with 10% FBS, 2 mM L-glutamine, 200 μM ascorbic acid, 50 mM β-mercaptoethanol, 30 mg/L penicillin, and 75 mg/L streptomycin. From 7 to 10 days of culture, 800 nM ICI182780 was added to the StemPro-34-based medium. At 11 days of culture, the culture medium was changed to StemPro-34-based medium without ICI182780	Mice	Production successful offspring; These findings have important implications in various areas including mammalian gametogenesis, genetic and epigenetic reprogramming, biotechnology, and medicine	Luo et al. (2021)
FGSCs were isolated and purified from ovaries collected from 5-day-old pou5f1/GFP transgenic mice × C57BL/6 F1 hybrid mice				
BXS0115 hiPSC line	STO-conditioned medium (Glasgow Minimum Essential Medium [GMEM] with 13% KSR and 1× non-essential amino acids, sodium pyruvate, and GlutaMax), supplemented with 100 ng/mL stem cell factor (SCF), 50 μg/mL ascorbic acid, and 25 µM 2-mercaptoethanol	Human	Provide unique opportunities for studying human ovarian biology and may enable the development of therapies for female reproductive health	Pierson Smela et al. (2023)
	GK15 medium (GMEM, 15% KSR, with 1× GlutaMax, sodium pyruvate, and non-essential amino acids) supplemented with 10 mM Y-27632, 0.1 mM 2-mercaptoethanol, 1 μg/mL doxycycline, 100 ng/mL SCF, and 50 μg/mL primocin			
	Alpha Minimum Essential Medium, 10% KSR, 55 µM 2-mercaptoethanol, 500 ng/mL doxycycline, and 50 μg/mL primocin			
Human ovarian samples	AdDE+++^a^ supplement with 50% v/v Wnt3aCM, Noggin, Rspo1, B27 (50x), 500 mM N-Acetylcysteine, Primocin, 1M Nicotinamide, 5 mM A83-01, 100 μg/mL Fgf10, 75 μg/mL Heregulinβ-1, 100 mM Y27632, 500 μg/mL EGF, 10 mM Forskolin, 250 μg/mL Hydrocortisone, 100 µM β-Estradiol	Human	NGS Wnts offer a unified organoid expansion protocol and a laboratory “tool kit” for dissecting the functions of Fzd subtypes in stem cell biology	Miao et al. (2020)
Human ovarian cancer, fallopian tube and ovarian surface epithelium	AdDE+++^a^ supplement with WNT (CM), Noggin, Rspo1, B27 (50x), 500 mM N-Acetylcysteine, Primocin, 1M Nicotinamide, 5 mM A83-01, 100 μg/mL Fgf10, 75 μg/mL Heregulinβ-1, 100 mM Y27632, 500 μg/mL EGF, 10 mM Forskolin, 250 μg/mL Hydrocortisone, 100 µM β-Estradiol	Human	Drug-screening assays; *in vivo* drug-sensitivity assays	Kopper et al. (2019)
FGSCs isolate from ovaries of 1–3 dpp female mice	GK15+ RA medium (Glasgow MEM supplemented with 15% Knockout serum replacement, 1.5 μM retinoic acid, 2 mM Glutamax, 1 mM MEM non-essential amino acids, 1 mM Sodium Pyruvate, 100 mM β-mercaptoethanol, 20 ng/mL EGF, 10 ng/mL bFGF, 10 ng/mL GDNF, 10 ng/mL LIF, and 100 IU/mL Penicillin-streptomycin). α-MEM-based medium (α-MEM supplemented with 2% FBS, 2 mM Glutamax, 100 μM ascorbic acid, 20 ng/mL EGF, 50 mM β-mercaptoethanol and 100 IU/mL penicillin-streptomycin) and StemPro-34-based medium (StemPro34 SFM supplemented with 10% FBS, 2 mM Glutamax, 100 μM ascorbic acid, 20 ng/mL EGF, 50 mM β-mercaptoethanol, 800 nM ICI182780, 100 IU/mL penicillin-streptomycin)	Mice	Basic research; pre-clinical drug screening	Wang et al. (2023a)
FGSCs isolate from ovaries of 1–3 dpp female mice	GK15+ RA medium (Glasgow MEM supplemented with 15% Knockout serum replacement, 1.5 μM retinoic acid, 2 mM Glutamax, 1 mM MEM non-essential amino acids, 1 mM Sodium Pyruvate, 100 mM β-mercaptoethanol, 20 ng/mL EGF, 10 ng/mL bFGF, 10 ng/mL GDNF, 10 ng/mL LIF, and 100 IU/mL Penicillin-streptomycin)	Mice	Establish a culture system that effectively expands FGSCs *in vitro*	Wang et al. (2023b)
Ovarian, peritoneal high-grade serous carcinomas, carcinosarcoma	AdDE+++^a^ supplement with WNT (CM), Noggin, Rspo1, B27 (50x), 500 mM N-Acetylcysteine, Primocin, 1M Nicotinamide, 5 mM A83-01, 100 μg/mL Fgf10, 75 μg/mL Heregulinβ-1, 100 mM Y27632, 500 μg/mL EGF, 10 mM Forskolin, 250 μg/mL Hydrocortisone, 100 µM β-Estradiol	Human	Drug development; personalized medicine applications	Phan et al. (2019)

^a^ AdDE+++:Advanced DMEM/F12 containing 1x Glutamax, 10 mM HEPES, and Pen Strep.

## 6 Summary and outlook

In conclusion, the ovarian organ function reconstruction techniques *in vitro* rebuilding technique can offer a fresh perspective on the study of ovaria-related disorders and fertility preservation in patients with cancers and POF. Future development direction in fertility preservation would focus on IVC, female GCs induction from PSC *in vitro*, artificial ovary construction, and ovaria-related organoids construction, all of which have promising futures. However, there are still problems to be resolved, including how to increase the primordial follicle maturation rate, establish a productive culture system with 3D technology, maintain the viability of follicles, build artificial human ovaries, and induce hPGCLCs to undergo meiosis. The construction of ovarian organoids is still a new method for ovarian reconstruction. However, it is extremely difficult to obtain human ovarian stem cells, so it is necessary to establish a single culture system that can efficiently expand FGSCs and maintain the differentiation of FGSCs into oocytes, and effectively improve the IVM rate of oocytes by combining with IVC technology. Organoid construction of ovarian cancer provides ideas for the study of the pathogenesis, drug screening and individualized therapy, gene function and immunotherapy of ovarian cancer. Meanwhile, the construction of tumor microenvironment of patient-derived tumor organoids should be further increased to reflect the specific situation more truly. Despite the numerous challenges, it is reasonable to assume that with the continued development of tissue engineering, new replacement options will eventually appear to offer new solutions to solve human reproductive problems like fertility preservation and ovarian diseases.
